# The effect of vection on the use of optic flow cues

**DOI:** 10.1098/rsos.250364

**Published:** 2025-07-23

**Authors:** Meaghan McManus, Katja Fiehler

**Affiliations:** ^1^ Psychology, Justus Liebig University Giessen, Giessen, Hessen, Germany

**Keywords:** vection, optic flow, self-motion, virtual reality

## Abstract

When we move objects move past us in a relative pattern of motion referred to as optic flow. Modulations in optic flow can impact both our perception of self-motion (e.g. perceived distance travelled) and our feeling of self-motion, referred to as vection (e.g. speed of self-motion). The perception and feeling of self-motion have so far been studied independently, leaving open whether and how the two relate to each other. In the current study, stationary participants performed a self-motion task in virtual reality where they moved to previously indicated distances using constant velocity optic flow. The perception of self-motion was measured as the ratio between the distance to travel and the distance travelled, where stopping sooner indicates that the optic flow cues were more effective in creating the perception of self-motion. Vection experience was measured via a questionnaire. When participants felt vection, there was a correlation between stopping distance (reflecting the perception of self-motion) and the felt speed of vection (reflecting the feeling of self-motion), i.e. the faster participants felt they were moving the sooner they stopped. These results show that the perception and feeling of self-motion are linked and that treating the two concepts independently can lead to misinterpretations.

## 1. Introduction

When we move through the world, the objects in our environment move past us in the opposite direction in a pattern of relative motion that is referred to as optic flow [1]. When we view a large field of optic flow this is typically because we are moving and so our perception of self-motion (in that we have interpreted the optic flow as being consistent with or indicating that we are moving through an environment) is coupled with a feeling of self-motion (a feeling of movement; a qualia), such as during walking. When walking forward we have both the perception that we are moving forward due to objects moving past us and we also have a feeling of motion. In this way, the perception and the feeling of self-motion are related. Variations in optic flow cues, such as the pattern of optic flow [2,3] and location of optic flow in the visual field [4], affect our perception of self-motion. Variations in optic flow, such as the speed of the optic flow [5] and the direction of motion [6], can affect the feeling of self-motion. In the present study, we investigated the relationship between our perception of self-motion and the feeling of self-motion when viewing the same optic flow stimulus, and expected that changes in one would affect the other.

### 1.1. The perception of self-motion

Optic flow is generated either when we move through our environment or if the environment moves past us and can be used to keep track of our location in the environment [7–9]. The effectiveness of optic flow cues in generating the perception of self-motion through an environment can be measured via move-to-target tasks where a stationary person must use optic flow cues to visually move until a previously indicated distance is reached [7]. The ratio between the distance the participant was supposed to travel and the distance they did travel (defined as gain) provides information on the effectiveness of the optic flow cues in generating the perception of self-motion through that environment. For instance, a participant would see a target 10 m in front of them. The target is then removed, and the person views optic flow while they are stationary. The optic flow provided is consistent with moving forwards. After the participant perceives that they have travelled 10 m they would stop the motion. If the participant stops before the previously seen target location, e.g. at 8 m (gain greater than 1), it is taken as evidence that the optic flow cues were more effective in generating the perception of self-motion than if they had to travel further than the previously seen distance, e.g. 12 m (gain less than 1). Our perception of self-motion has been found to vary based on where in the field of view the visual motion cues are present [4] and which type of motion is viewed [3,9–11]. The effectiveness of optic flow cues in generating the perception of movement through an environment might also be influenced by changes in sensory weighting [8], where an increase in the weight given to visual cues might lead to a higher gain (travelling less far).

### 1.2. The feeling of self-motion (vection)

Typically, when we are viewing a large field of optic flow it is because we are moving and so we also experience a feeling of self-motion. This feeling of self-motion, however, can also occur when we are not moving but simply viewing large-field optic flow. For example, while sitting in a train most of us have experienced the illusion that occurs when the train next to us moves and we think it is our train that is moving. Once the other train leaves the station, we realize we have not actually gone anywhere. This illusionary feeling of self-motion induced by optic flow is referred to as vection [12,13]. Vection has been defined in many different ways (see [14] for a review on vection). There is the way it is defined above, as (i) a visual illusion that occurs when a stationary person views optic flow [12,13]. (ii) An illusion that can arise from visual or non-visual cues, as this feeling can also be induced through things like moving auditory cues [15]. (iii) The subjective experience of self-motion mediated by visual cues. In this case, the participant’s body might also experience motion, such as when walking on a treadmill (e.g. [16]). And lastly, (iv) a conscious subjective experience of self-motion [17] or the ‘*absolute sensation of motion’* [18].

When a stationary person is viewing optic flow and interprets this as self-motion through an environment this interpretation can be accompanied by a feeling that they are moving or not. In our paper, when we refer to vection we are exclusively referring to this feeling that can occur which is predicated on first interpreting optic flow cues as consistent with movement through an environment. In that case, our definition of vection aligns most closely with the first and fourth definition with the emphasis that we are referring to the feeling of self-motion.

Vection is influenced by factors such as the size of the field of view [12], the direction of the optic flow [6], gravity [19,20] and body tilt [21]. Measures of vection include its magnitude or intensity (the strength of the feeling; [22,23]), its onset (when the illusion starts; [13,24]), its duration (how long the feeling lasts for; [25]), and the felt speed of movement; [12,19]. Higher order factors also play a role in the feeling of self-motion [23,24,26]. Previous research has shown that the plausibility of a situation and personality traits [24] can affect vection where participants who experience the same visual cues can experience vection differently. Similarly, vection is influenced by the perceived scene context [23,26], where when the scene is more naturalistic vection is enhanced [23]. Vection has also been found to enhance spatial updating and perspective taking [15] (see also [27] for a discussion on how the presence (or lack thereof) might have impacted spatial updating). For example, Riecke & colleagues [15] asked blindfolded participants to imagine the room they were in from different perspectives and point to the locations of objects from that perspective. Participants showed better performance when rotational auditory cues (a rotating sound field) and biomechanical feedback induced the feeling that they had rotated to the new perspective.

### 1.3. Are the perception and feeling of self-motion related?

As outlined above, both the perception of self-motion and the feeling of self-motion can occur when viewing optic flow. The perception of self-motion refers to our understanding that we are moving when viewing optic flow. We can use optic flow cues to determine how far we have moved through the environment, such as during the move-to-target task. The feeling of self-motion refers only to the feeling that we are moving when viewing optic flow (vection). It appears logical that the perception and the feeling of self-motion should be related.

**Figure 1.**
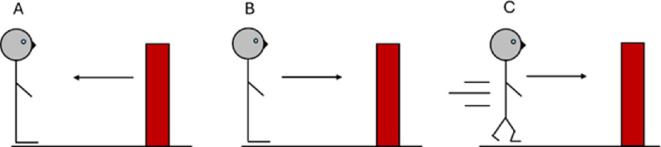
The different ways a person might interpret optic flow. Panel (A) depicts a stationary person perceiving the world moving past them. Panel (B) depicts the situation where the person perceives the world as stationary and so interprets the optic flow as self-motion (i.e. they have a perception of self-motion), but they do not experience a feeling of self-motion. In this case, the person can still use the optic flow cues to keep track of the distance moved despite the lack of a feeling of self-motion. This is similar to when playing a first-person video game. Panel (C) depicts a person perceiving themselves as moving forward while also experiencing vection. The person perceives the world as stationary and so interprets any optic flow as self-motion while also feeling like they are moving (i.e. they have both a perception of self-motion and a feeling of self-motion). It is possible that a person could experience a feeling of self-motion without the perception of self-motion, such as during vertigo, though this situation would be unlikely in the current experiment due to the optic flow cues provided.

In support of this notion, previous studies that have introduced a spatial jitter to optic flow (e.g. the optic flow oscillates up and down or side to side) have found an enhancement to the feeling of vection where vection magnitude is increased [13,28,29], while other studies have found an enhancement to our perception of self-motion (higher gain) [3,11] compared with no jitter. Similarly, in [15], illusionary motion induced by auditory and biomechanical cues improved participants' pointing performance when imagining the room they were in from a different perspective. However, while a relationship between the feeling and perception of self-motion (figure 1C) appears logical, the perception of self-motion can occur without a feeling of self-motion where a person can visually understand that they are moving forward despite not feeling they are moving (such as when playing a first-person video game), see figure 1B. A person can also keep track of how far they have moved in an environment in the absence of a perception and feeling of self-motion, figure 1A. In this case, a person might perceive the world as moving past them while they are stationary.

It is possible then that the feeling of self-motion and our perception of self-motion might be unrelated. This is in line with the findings that while the speed of optic flow affects our feeling of self-motion where it is positively related to the felt speed of vection [5], the speed of optic flow does not appear to affect our perception of self-motion in that gain is not affected by optic flow speed [7], though see [10] for an alternative finding. Additionally, while the compellingness of vection and the felt speed of vection are enhanced during microgravity flight [19,20], there are no changes in our perception of self-motion [30]. These findings suggest that changes in the feeling of self-motion can occur without changes to the perception of self-motion. Similarly, changes in our perception of self-motion might not necessarily affect the feeling of self-motion. If we stop sooner when moving through an environment this would likely not retrospectively make us feel as if we had moved faster.

### 1.4. Current study

In the current study, we investigated if there was a relationship between changes in the perception of self-motion and the feeling of self-motion. The findings will help us to clarify whether and how the two concepts are linked to each other.

Participants performed a move-to-target task in virtual reality. After each trial they answered a questionnaire, presented in the same virtual reality environment, about any vection they might have experienced on that trial such as whether or not they experienced vection (with the option to select one of the three options described in figure 1), the compellingness of any vection experienced (how believable it felt that they were moving), the duration of the feeling, as well as the felt speed of their self-motion, and the perceived speed of the world around them. The participants’ performance on each trial of the move-to-target task (gain) was taken as a measure of their perception of self-motion through the environment on that trial. The measures of vection were taken as a measure of their feeling of self-motion on that trial. Because a participant cannot be made to experience vection, the analyses are correlational. We can look within trials when the participant did experience vection, or not, and see how the feeling and perception of self-motion are related.

We proposed that the feeling and perception of self-motion are related. We hypothesized (i) that when the participant experiences vection the gain from the move-to-target task will be positively correlated with the felt speed of self-motion. When a person feels themselves as moving faster they will have a higher gain (and so stop sooner) compared with when they feel themselves as moving more slowly despite viewing the same optic flow. While no relationship between the speed of optic flow and gain has been found [7] this study did not report vection experience. Differences in the felt speed of the self as well as experiencing vection or not could have obscured any effect. We further hypothesized (ii) that the compellingness of vection will be positively correlated with gain as this might impact the weight given to the feeling of vection. The duration of vection should also be positively correlated with gain (iii) as, if the feeling lasts longer, its effect will be compounded. Finally, we hypothesized (iv) that the perceived speed of the world will be positively correlated with gain when participants perceive the world is moving past them instead of perceiving themselves as moving.

## 2. Methods

### 2.1. Participants

Thirty participants (mean age = 23.50 years, SD = 3.63 years, 12 males, 17 female, 1 diverse) were recruited from the Justus-Liebig-University Gießen. A justification of the selected sample size is provided in §2.12 at the end of §2.8 below. One participant could not complete the task due to a computer issue, one participant withdrew due to illness, and one participant was stopped from participating as they did not comply with task demands (walking around the environment). This left us with 27 participants. The experiment was conducted in agreement with the Declaration of Helsinki (2013, except pre-registration of the study) and was approved by the local ethics committee of the Faculty of Psychology and Sports Science at the Justus Liebig University Giessen, Germany (Certificate # 2019-0003). All participants were naïve to the purpose of the study and were compensated at a rate of 1 academic credit per hour or 8 euros per hour. All participants provided written informed consent and reported normal or corrected-to-normal vision, no vestibular impairment and no history of seizures, fainting or migraines.

### 2.2. Apparatus

Stimuli were presented via an HTC Vive Pro Eye virtual headset (HMD). The HMD has a field of view that extends approximately ± 110° diagonally. The HMD screen has a 1400 × 1600-pixel resolution per eye and a 90 Hz refresh rate. Stimuli were created in Unity (version 2019.4.1241; Unity Technologies, Inc., San Francisco, CA, USA) and displayed via SteamVR (version 1.24.7). The experiment was run on a Razer Blade Laptop (Intel(R) Core (TM) i7-8750H CPU @ 2.20 GHz, 16 GB RAM, NVidia GeForce GTX1070 Max-Q GPU). The projection was stereoscopic and was actively linked to the position of the participant’s head. Therefore, distance cues were available from stereoscopic cues and motion parallax. Stereoscopic cues not only provide extra cues for interpreting distance but might also help to enhance vection experience [31].

### 2.3. Stimuli

**Figure 2.**
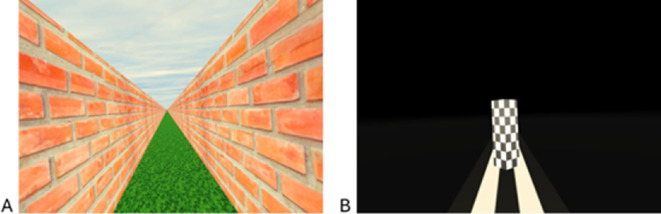
A screen capture of the virtual environments the participants saw in the headset. Panel (A) shows the hallway environment that was used during the presentation of visual motion. Panel (B) shows the dark environment that displayed the target object used when estimating the distance to travel.

Two virtual environments were used. One of the environments, the hallway environment, was a simulated virtual hall that consisted of two walls (3 m high and 510 m long) with a seamless brick texture and a floor (510 m long) which contained a seamless grass texture. The ceiling of the hallway contained a seamless cloud texture. A wall height of 3 m was chosen as it is within the standard range of wall height for a house. The length of 510 m was used as, based on previous experience using a similar task, it is more than twice the furthest expected distance that a participant might travel [8]. The textures had no repeating discernible features to prevent participants from being able to track their position. See figure 2A.

The second environment, the dark environment, consisted of only a floor (510 m long) with a black and white striped texture and a pillar (referred to as the ‘target object’; 2 m high × 50 cm × 50 cm) with a black and white checker pattern. The target object was rotated by 45° such that when the participant looked at it, they were looking at the target object’s corner. This was done to help increase the perception of depth. See figure 2B.

### 2.4. Tasks

To investigate the relationship between the feeling of self-motion and the perception of self-motion we first need a measure of each experience.

### 2.5. Move-to-target task

To get a measure of the participants’ perception of self-motion we applied a move-to-target task. Participants viewed the target object in a dark environment (figure 2B) at one of five distances from themselves (10, 20, 30, 40, 50 m), and then visually estimated their distance to the object. When ready participants left-clicked on the mouse held in their right hand. Instantaneously, the dark environment and target object were rendered off and the hallway environment in figure 2A was displayed. At the same time, stereoscopic optic flow consistent with forward motion started at a rate of 2 ms^−1^. The participant’s task was to stop the optic flow, using the right mouse button, when they felt they had reached the location of the previously viewed target object. The presentation of the target distances from the participant was randomized. The participants viewed the target object in the dark environment to limit their ability to line it up with texture cues in the hallway environment and use them as landmarks to travel to the respective target location instead of relying on the optic flow cues.

### 2.6. Questionnaire

In order to get a measure of the participants’ feeling of self-motion on a given trial we assessed the participants’ experience of vection on that trial using a questionnaire projected in the virtual environment. The questionnaire consisted of five questions. See table 1.

**Table 1.** The questions provided in the questionnaire along with the possible responses to that question. The participants selected the number that best represented what they experienced on that trial. Labels were provided below some of the numbers to help the participants to interpret the response.

topic	question	response	response	response	response	response	response
vection experience	what type of movement did you feel?	1 the world moved past me. I did not move	2 I perceived that I moved but felt no vection	3 I felt vection			
vection compellingness	how compelling was the vection?	0 I did not feel vection	1	2 vection was convincing	3	4 vection was very convincing	
vection duration	for how long did you experience vection?	0 I did not feel vection	1	2 for half the time	3	4 for the whole time	
world speed	how fast did the world feel like it was moving?	0 no motion	1 much slower than walking speed	2	3 walking speed	4	5 much faster than walking speed
self speed	how fast did you feel you were moving?	0 I did not move	1 much slower than walking speed	2	3 walking speed	4	5 much faster than walking speed

The participants selected a response using the VR controller. A small sphere was projected onto the surfaces the participants used as cursor to point to the respective response box. Participants used the trigger button to select their answer which caused the answer to turn magenta. They then confirmed their response by pressing the ‘Done’ button at the bottom. This started the next trial of the move-to-target task.

### 2.7. Procedure and design

Upon arriving at the lab participants were given a brief description of the task and a definition of vection using the example of sitting in a train at a train station. The participant was asked if they had ever experienced the phenomenon where the train next to them moves and they think they are the one moving. If they had experienced this, they were told that this illusionary feeling was referred to as vection. If they had not experienced this then the illusion was explained to them. Participant then signed the consent form and read the task instructions. Afterwards, they were provided with written instructions of the task. Following this, each question on the questionnaire was explained and open questions were answered. After, the participants were familiarized with the use of the headset and the VR controller.

The experiment started with five practise trials that were not included in the analysis. The first practise trial did not contain the target object and was used to practise the start and stop procedure (left to go, right to stop). The next four practise were used to practise the move-to-target task and so contained the target object. The target distances used in the practise trials differed from the experimental condition and were always presented in the same order (5, 15, 55 and 35 m). After completing the five practise trials, participants performed the experiment block in a self-paced manner. After each trial of the move-to-target task, the questionnaire appeared in the VR headset. After responding to the questions, the next trial started with the target object at the next randomly selected target distance from the participant. There was a mandatory break after 15 min where the participant had to take the headset off and sit down until they decided to continue the task. Participants could also stop the experiment at any given time. There were 50 trials in total (5 distances × 10 repetitions), however due to an error when saving the data the last trial was not saved so there was only 49 trials per participant. The experiment took an hour on average.

### 2.8. Data analysis

#### 2.8.1. Pre-processing

First, we removed mistrials (stop distance = 0 m). We removed 11/1323 (0.83%; 27 participants with 49 trials each) trials. Then for each trial the gain was calculated (target distance/travel distance). The trials for each person were then divided up based on the response to the vection experience question. The first group of trials, the V0 data, contained the data from a trial where the participant responded with a 1 on the vection experience question (‘World moving’, figure 1A), the second group of trials, the V1 data, contained the data from a trial where the participant responded with a 2 on the vection experience question (‘Self moving but no vection’, figure 1B), and the third group of trials, the V2 data, contained the data from a trial where the participant responded with a 3 on the vection experience question (‘Vection’, figure 1C).

We then performed an outlier analysis for each vection experience group. If the gain was ± 2.5 standard deviations away from the mean gain of that vection experience group this trial was removed. This resulted in the removal of 6/262 data points (2.29%) from V0, 9/420 data points (2.14%) from V1 and 20/630 data points (3.17%) from V2. See the electronic supplementary material, table S1 for the number of trials per participant in each vection group.

### 2.9. Variations in vection experience over the course of the experiment

Additionally, it is possible that vection experience might vary over the course of the experiment as participants become more familiar with the tasks, e.g. more experience of vection later than earlier in the experiment. If this does occur, then the data from the no vection group (V0) might be more likely to occur early in the experiment and the vection group (V2) later in the experiment. If there are, then variations in gain across the experiment could explain any effect found. To investigate potential changes in the occurrence of vection over time, we compared the distribution of trial number for each vection group using three 2-tailed Mann–Whitney U tests. The Mann–Whitney U test was selected as the data (trial number) were ordinal. We did not control for multiple comparisons.

### 2.10. Questionnaire responses and gain

For each person within a vection experience group the average gain, median vection compellingness, duration, felt speed of self motion, and perceived speed of world motion were calculated using R (version 2021.09.1). We used medians as the responses to the vection questionnaire were ordinal. For example, for participant 30, there were 12 trials where the participant reported no vection (V0). These were used to calculate their average gain and the median vection scores for the V0 data. See the electronic supplementary material, tables S2–S4 for the number of trials per participant per visual condition, and the average gains and the median vection responses calculated per vection group, per participant.

### 2.11. Relationship between gain and the median vection questionnaire responses

In order to determine if there is a relationship between changes in the feeling of self-motion and our perception of self-motion we performed a series of 2-tailed Spearman’s correlations between gain and the responses to each of the questions on the vection experience questionnaire. The correlational analyses were performed in IBM SPSS (Version 30.0.0.0). This was done for each of the three different vection experience groups (world moving, self moving and vection). Spearman’s correlations were run as the questionnaire responses were ordinal but the gains were continuous.

For vection groups V0 and V1 no correlations between the compellingness of vection and gain, and the duration of vection and gain could be calculated because fewer than three participants in each group had a median response other than 0 (‘I did not feel vection’). Additionally, for the V0 group, for the reported felt speed of self, only six participants had a median value of other than 0 (‘I did not move’) and so no correlation was calculated for this condition as they would be unreliable. This was expected as participants who do not feel vection should also not report any compellingness, duration or experience of self-motion. For the specific hypotheses, see §1.

### 2.12. Determining sample size

As our experimental design does not allow for pre-determining the sample size for each of our three vection groups, we did not perform a standard sample size estimation (e.g. [32–34], see also [35]). Instead, we based our sample size calculations on a literature review including studies on both self-motion perception (i.e. using gain as a measure) and the relationship between vection and other measures (e.g. personality traits [24], perceived velocity [36], and presence [23]). We considered the number of participants used in different studies as well as the reported effect sizes and correlation coefficients. Previous research investigating variations in gain on a move to target task have typically used sample sizes around 20 participants [7–11,37]. The reported effect sizes were moderate (partial eta squared ca. 0.3) when comparing differences in gain or distance travelled [8,37]. For the studies investigating the relationship between vection and other measures, samples sizes were typically around 17 with the reported correlation coefficients being moderate to high (ca. 0.6) [23,24,36].

Based on these studies we concluded that a sample size of 20 participants would be sufficient for our study. To account for some more variability in our data and due to the fact that we could not control the number of participants in each group, we decided to collect data from 30 participants to ensure sufficient power for our data analyses.

## 3. Results

### 3.1. Variations in vection experience over the course of the experiment

To determine if the occurrence of vection differed across the course of the experiment we performed three 2-tailed Mann–Whitney U tests comparing the distributions of trial number in each combination of the vection groups. The V0 group (mean trial number = 23.61, SD = 14.17) did not significantly differ from the V1 group (mean = 23.36, SD = 14.01), *U =* 52096, *p* = 0.832. The V0 group did not differ from the V2 group (mean = 25.56, SD = 14.12), *U =* 81091, *p* = 0.370 and the V1 group did not differ from the V2 group (*U =* 131552, *p* = 0.180).

Overall, there appear to be no systematic differences in the occurrence of vection between the different vection groups.

### 3.2. Perception of self-motion

**Figure 3.**
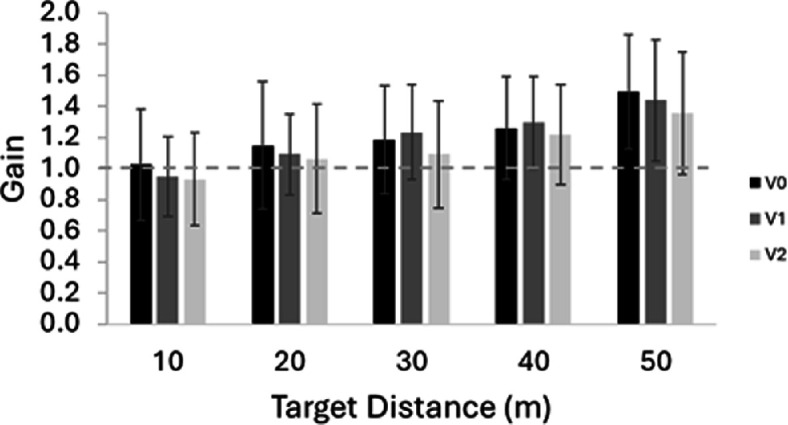
The average gain plotted for each target distance for each vection group. Error bars are ± standard deviations. Standard deviation was used in order to best show the variation among the participants.

To examine if the participants were able to accurately move to the different distances, we plotted the average gain (target distance/travel distance) for each target distance for each vection group. The gain measures how effective the optic flow cues were at creating the perception of self-motion through the environment. A gain of 1 indicates that participants accurately moved to the target distance. A higher gain (undershooting the target distance) indicates a more effective perception of self-motion through the environment as less optic flow is needed to perceive that they have reached the target distance. A lower gain (overshooting the target distance) indicates a less effective perception of self-motion through the environment as participants needed to travel further (need more optic flow) to perceive they had reached the target distance. See figure 3. In general, the results show that the participants were fairly accurate but that they tended to undershoot the target distance more the further the target, indicating a more effective perception of self-motion through the environment for further distances.

### 3.3. The relationship between perception and feeling of self-motion

**Figure 4.**
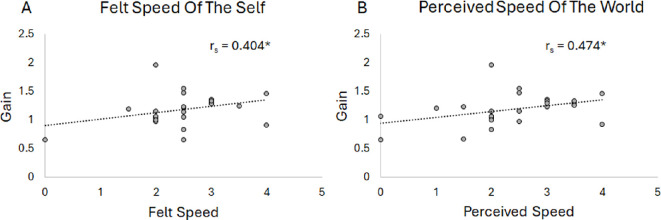
Scatterplots of the relationship between the felt speed of the self and the average gain (Panel A) and the perceived speed of the world and the average gain (Panel B) for the vection response group (V2). A regression line was fitted to all data (dotted line) and the correlation value is given. An asterisk indicates the correlation is significant (*p* < 0.05).

When participants experienced vection (V2), there was a significant moderately positive correlation between gain and the felt speed of the self, inline with Hypothesis 1. This indicates that as the felt speed of the self increased, the gain also increased, *r*_s_ = 0.404, *p* = 0.045, see figure 4A. For participants who perceived self-motion but did not experience vection (V1), no significant correlation was found between the felt speed of the self and gain *r*_s_ = −0.133, *p* = 0.535. For participants who did not feel any self-motion (V0) not enough participants responded with a value greater than 0, so no reliable correlation could be calculated.

For the relationships between vection compellingness and gain (Hypothesis 2) and vection duration and gain (Hypothesis 3), none of the correlations reached significance when participants experienced vection (V2), compellingness: *r*_s_ = −0.08, *p* = 0.692 and duration: *r*_s_ = −0.03, *p* = 0.874. For the V0 and V1 groups not enough participants responded that they felt a sense of compellingness or duration of vection so no reliable correlations could be calculated.

For the relationship between the perceived speed of the world and gain (Hypothesis 4), when no vection was experienced (V0), there was no significant correlation between the perceived speed of the world and gain, *r*_s_ = −0.206, *p* = 0.412. When participants perceived self-motion but did not experience vection (V1), no significant correlation was found between the perceived speed of the world and gain, *r*_s_ = 0.164, *p* = 0.445. However, when participants did experience vection (V2), there was, unexpectedly, a significant moderately positive correlation between the speed of the world and gain where, as the perceived speed of the world increased the gain increased too, *r*_s_ = 0.474, *p* = 0.017, see figure 4B.

Overall, when participants experienced vection there was a relationship between the perception of self-motion and multiple measures of the feeling of self-motion (felt speed of the self, and perceived speed of the world). As the felt or perceived speed increased gain also increased, meaning that the participants stopped sooner on the move-to-target task. A higher gain indicates a more effective perception of self-motion through the environment.

### 3.4. Exploratory analysis comparing perceived speed of the world and felt speed of the self

**Figure 5.**
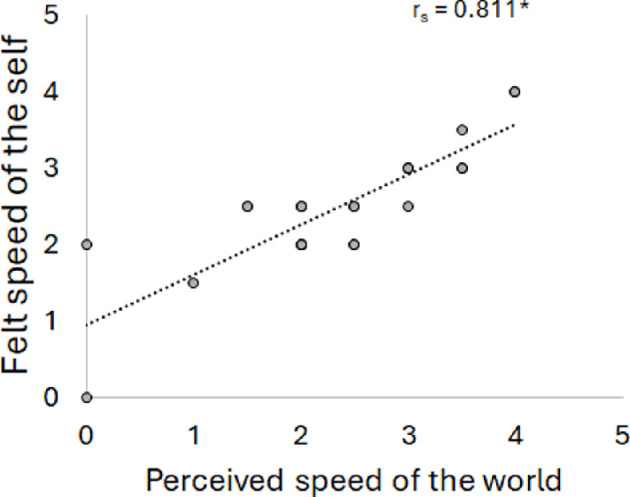
Scatterplot of the relationship between the perceived speed of world and felt speed of self for the vection response group (V2). A regression line was fitted to all data (dotted line) and the correlation value is given. An asterisk indicates the correlation is significant (*p* < 0.05).

Given the unexpected finding of the relationship between gain and the perceived speed of the world in the V2 group, we performed a post hoc correlation to determine the relationship between the perceived speed of the world and the felt speed of the self for the V2 group. We found a strong, positive correlation, *r*_s_ = 0.811, *p* =<0.001 indicating the faster they felt the speed of their self, the faster they perceived the speed of the world, figure 5.

## 4. Discussion

In this study, we examined the relationship between the perception of self-motion and the feeling of self-motion. When visually moving through the environment participants had a tendency to stop before the target, i.e. they produced a shorter movement distance, regardless of how they felt or perceived their movement through the environment. This effect increased with increasing target distance. This is consistent with previous research on self-motion perception [4,9] where the travel distance can be modelled as a leaky spatial integrator [7].

While it is known that optic flow cues can affect our perception of self-motion [4,9] and the feeling of self-motion (e.g. [5,12]), no study has looked at how our perception and the feeling of self-motion are linked to each other. We had hypothesized a relationship between the two when viewing the same optic flow. Specifically, when participants experience vection there should be a relationship between the felt speed of self-motion and gain (Hypothesis 1) where gain is a measure of our perception of self-motion. We also hypothesized that when experiencing vection, the compellingness (Hypothesis 2) and duration (Hypothesis 3) of the feeling of vection should lead to a higher gain. Finally, we had expected the perceived speed of the world to be positively related to gain when participants did not experience vection (Hypothesis 4). Overall, we found a moderate positive correlation between the felt speed of the self and gain but only when participants experienced vection. In support of Hypothesis 1, our findings suggest a moderate positive relationship between our perception and feeling of self-motion. Interestingly, we also found a strong positive correlation between the felt speed of the world and gain but only when participants experienced vection. This is counter to Hypothesis 4. The felt speed of the self and gain and the felt speed of the world and gain were not related in any other vection group. The other measures of vection were not found to be related to gain in any vection group (Hypotheses 2 and 3).

### 4.1. The effect of the felt speed of self-motion on gain

When participants experienced vection, the perception of self-motion (gain) was positively related to the speed at which participants felt they were moving. Despite all participants viewing the same constant velocity through the same visual environment, on trials where participants experienced vection, the gain varied with the felt speed of the self such that participants stopped sooner (high gain) when they felt they were going faster and travelled further (low gain) when they felt they were going slower. While the analysis was correlational it is unlikely that arbitrarily stopping sooner causes participants to feel, retrospectively, like they had travelled faster. Instead, the results indicate that the felt speed of self-motion led to the changes in stopping distance. This suggests that not only do differences in the speed of optic flow lead to changes in the felt speed of vection [5] but the felt speed of vection can also lead to changes in how we use optic flow cues to control our movement through an environment (our perception of self-motion). While our correlation was performed on a relatively small set of data, we believe the effect is robust given that our sample size is inline with other studies investigating similar topics, and the effect found is moderately sized and in line with the expected effect.

These findings help to bring together two topics in self-motion perception which have up until now been almost exclusively studied separately [15,27]. The results presented here show that vection does have an impact on behaviour meaning that it is likely that vection is not just an epiphenomenal experience but might serve some function (see [14] for a discussion on the functional significance of vection). Indeed, the experience of vection while viewing optic flow has also been found to lead to changes in brain activation with increases in activity in visual areas related to motion coherence and self-motion (MT+ and V6 respectively), vestibular (PIVC) and multisensory (VIP) brain areas compared with when people are viewing optic flow but not feeling vection [38].

While our paper has focused on distance travelled as a measure of the perception of self-motion, other measures such as heading and path integration might also be impacted by the feeling of self-motion [15,27]. In future studies on the feeling of vection or the perception of self-motion both measures should be taken in order to best interpret any results. The findings here might also help to shed light on surprising findings in the literature such as, despite there being an increased sensitivity to vection in microgravity, [19,20] there appear to be no changes in gain [30]. However, our results leave some open questions. Why when viewing the same optic flow in the same environment do different people, or even the same person on two trials, perceive their speed differently? This could be related to where the participant looks during the trial [39] or perceived eye height in the environment which would lead to different estimations of scale [40]. What is happening internally when the participants interpret the optic flow cues as consistent with self-motion in the absence of vection? We assume that the participants are keeping track of how far they think they have moved and how far they still have to move, whether this is by imagining they are moving, relying on a sense of timing, or simply integrating the information provided by the optic flow cues. However, it is clear that the participants are able to reliably move to the different distances and so must be perceiving themselves as moving through the environment in some way.

### 4.2. The effect of the perceived speed of the world on gain

If we feel we are moving, then we are interpreting at least some of the optic flow as self-motion. Any optic flow that is not interpreted as self-motion would then have to be interpreted as world (or object) motion. The amount of motion attributed to self-motion versus any ‘leftover’ world motion is a measure of vection saturation. If all perceived motion is solely interpreted as self-motion this would indicate complete vection saturation [41–43]. Interestingly, when participants in this experiment experienced vection, the speed they perceived the world to be moving past them also affected gain. This suggests that our participants did not have full vection saturation; if they did have full vection saturation, then they would perceive the world as static. Our exploratory analysis also revealed a very strong, positive relationship between the felt speed of the self and the perceived speed of the world when participants were experiencing vection. When participants felt they were moving quickly they also perceived the world was also moving past them quickly.

For our two vection groups that did not experience vection, there was no relationship between world motion and gain. While this might appear surprising, previous work has not found a relationship between the speed of optic flow itself and gain [7], i.e. the participants moved similarly to the different target distances regardless of the speed of the optic flow. The present results support this finding, but only for participants who do not feel vection. Participants who experienced vection were sensitive to changes in the speed of the optic flow.

### 4.3. The different measures of vection and gain

We had hypothesized that the gain would positively correlate with both the compellingness and duration of vection when participants experienced vection. If the vection experienced was more compelling it might lead to an increase in the weight given to the feeling of self-motion. Additionally, if the feeling lasted longer, we had hypothesized this would compound any other effect. However, we found neither a relationship for compellingness nor for duration. In a study by [36], they correlated vection latency, duration and magnitude with the perceived velocity of the display of moving dots that they were viewing. Overall, they tended to find that the measures of vection were not correlated with the perceived speed of the dots with only 18 out of 48 (16 participants × 3 measures) correlations reaching significance, though when a correlation was found it tended to be a strong correlation with correlation coefficients around 0.65 (positive or negative depending on the measure). In our study, if participants experienced some form of vection for any duration, then depending on how they interpreted their own felt speed in that instant this led to changes in gain.

### 4.4. Optic flow versus timing

When driving to your friend’s house down the street you do not have to rely solely on optic flow cues. You know that your friend lives in a particular house, what that house looks like, and what the neighbouring house looks like. This tells you when to stop driving, not when a certain amount of optic flow cues has passed. While optic flow cues are produced during self-motion one only needs to monitor them to ensure they are not driving too fast since other cues will tell you when you have driven far enough. In the current experiment there were no distinct visual cues like houses on a street. Instead, participants only had the textures on the surfaces to keep track of how far they had moved. This could increase participants’ reliance on optic flow cues to monitor the distance travelled. However, it is possible that instead of continuously using optic flow cues, participants were instead relying on a time estimate. In this case, the participant would estimate the distance to the target object and the speed they are moving based on both the optic flow cues provided (e.g. are they moving consistently, accelerating or is there a jitter) and the feeling of self-motion. Then they could calculate an estimate of when they should stop and can ignore the rest of the optic flow. While this remains a possibility, we do not believe it affects the interpretation of this study as participants still need to combine both the optic flow cues and the feeling of self-motion. However, it would help to explain why the duration of vection experience was not related to gain.

## 5. Conclusion

While the effect of optic flow on the perception of self-motion and on the feeling of self-motion has previously been established, there is limited research on how the two impact each other. Based on our results, we found a positive correlation between the speed participants felt they were moving and the distance they travelled. When participants felt they were moving faster they travelled shorter distances (higher gain) compared with when they felt they were going slower, despite viewing the world moving with a fixed constant velocity. Overall, it appears that the perception and feeling of self-motion are linked. It will be important for future studies investigating self-motion to include a measure of both in order to reduce the likelihood of misinterpretations. This is particularly true for studies investigating the perception of self-motion as travel distance appears to be influenced by vection experience.

## Data Availability

All data, analysis code and supplementary data are openly available at the project’s Open Science Framework page [44]. Supplementary material is available online [45].
